# MicroRNAs 218a-5p, 219a-5p, and 221-3p regulate vestibular compensation

**DOI:** 10.1038/s41598-017-09422-8

**Published:** 2017-08-18

**Authors:** Mun Young Chang, Sohyeon Park, Jun Jae Choi, Young-Kook Kim, Myung-Whan Suh, Jun Ho Lee, Seung Ha Oh, Moo Kyun Park

**Affiliations:** 1 Department of Otorhinolaryngology-Head and Neck Surgery, Chung-Ang University College of Medicine, Seoul, 06973 Republic of Korea; 2 Department of Otolaryngology-Head and Neck Surgery, Seoul National University Hospital, Seoul National University College of Medicine, Seoul, 03080 Republic of Korea; 3 Department of Biochemistry, Chonnam National University Medical School, Gwangju, 61186 Republic of Korea

## Abstract

Unilateral vestibular deafferentation (UVD) interrupts afferent signals from one side, resulting in an imbalance of the resting activity between bilateral vestibular nuclei. Vestibular compensation is the process of balancing the resting activity to reestablish homeostasis. Here, we investigated microRNAs (miRNAs) that regulate vestibular compensation using the Sprague–Dawley rat. After determining the progression of vestibular compensation following UVD, microarray analysis was performed and nine miRNAs were selected as candidates. Following validation by quantitative reverse transcription-PCR, three miRNAs remained. We assessed the effect of these miRNAs on vestibular compensation using miRNA oligomers. We compared the results of the rotarod test and 5-bromo-2′-deoxyuridine immunohistochemistry following UVD between the control group and the groups in which the candidate miRNA oligomers were administered. Administration of miR-218a-5p, 219a-5p, and 221-3p oligomers significantly affected vestibular compensation. Target pathway analysis of these miRNAs supported our results. Our findings suggest that the miRNAs 218a-5p, 219a-5p, and 221-3p regulate vestibular compensation.

## Introduction

Normal vestibular function, which is essential for balance, depends upon symmetrical afferent signals from vestibular organs in the inner ears. Afferent signals converge upon the brainstem vestibular nuclei and affect oculomotor and postural reflex function. Unilateral vestibular deafferentation (UVD) induces complete or partial interruption of the afferent signals from one side leading to severe imbalance of the resting activity between the ipsilesional and contralesional vestibular nuclei neurons^1^. This imbalance impairs oculomotor and postural reflex function and causes severe dizziness^2, 3^. These symptoms disappear as vestibular compensation is achieved. However, in some patients with UVD, proper vestibular compensation is not achieved and the dizziness continues. The prevalence of dizziness caused by vestibular dysfunction has been reported to range from 1.8% to 35.4%^4–6^. Therefore, identifying the mechanism underlying vestibular compensation may be useful in the management of patients with UVD.

Vestibular compensation is a process in which the firing rates of bilateral vestibular nuclei neurons are rebalanced, resulting in bilateral excitability homeostasis. It is thought that vestibular compensation is achieved mainly through the modulation of inhibitory systems in the medial vestibular nucleus (MVN)^7–9^. Vestibular compensation progresses rapidly and the initial symptoms caused by UVD improve over days to weeks^10^. Vestibular compensation is a phenomenon typical of rapidly progressive neuronal plasticity^11^, which is an attractive model for neuronal plasticity research.

MicroRNAs (miRNAs) are small non-coding RNAs that post-transcriptionally regulate gene expression through translational repression or degradation of mRNA^12^. It is now accepted that the expression of most genes is modulated by miRNAs. Sequentially acting miRNAs are involved in most biological processes. Furthermore, it has been found that miRNAs are involved in the pathogenesis of several diseases, including neurological and cardiovascular diseases, cancer, and diabetes mellitus and miRNAs are now being recognized as potential therapeutics^13–15^.

miRNAs are abundant in the brain and play a crucial role in the development and function of the neuronal network, including neurogenesis, synaptogenesis, and regulation of morphogenesis^16–18^. It is reasonable to postulate that miRNAs play an important role in vestibular compensation. To the best of our knowledge, however, miRNAs involved in vestibular compensation have not yet been reported. In the present study, we identified miRNAs that regulate vestibular compensation following UVD. The study consisted of five steps: determination of the progression of vestibular compensation following UVD, microarray analysis, quantitative reverse transcription (qRT)-PCR validation of miRNA expression, validation using candidate miRNA oligomers, and finally target pathway analysis of the candidate miRNAs. This study will help lay the foundation for future studies of the functional mechanism of vestibular compensation and neuronal plasticity.

## Results

### Vestibular compensation time course

**Figure 1:**
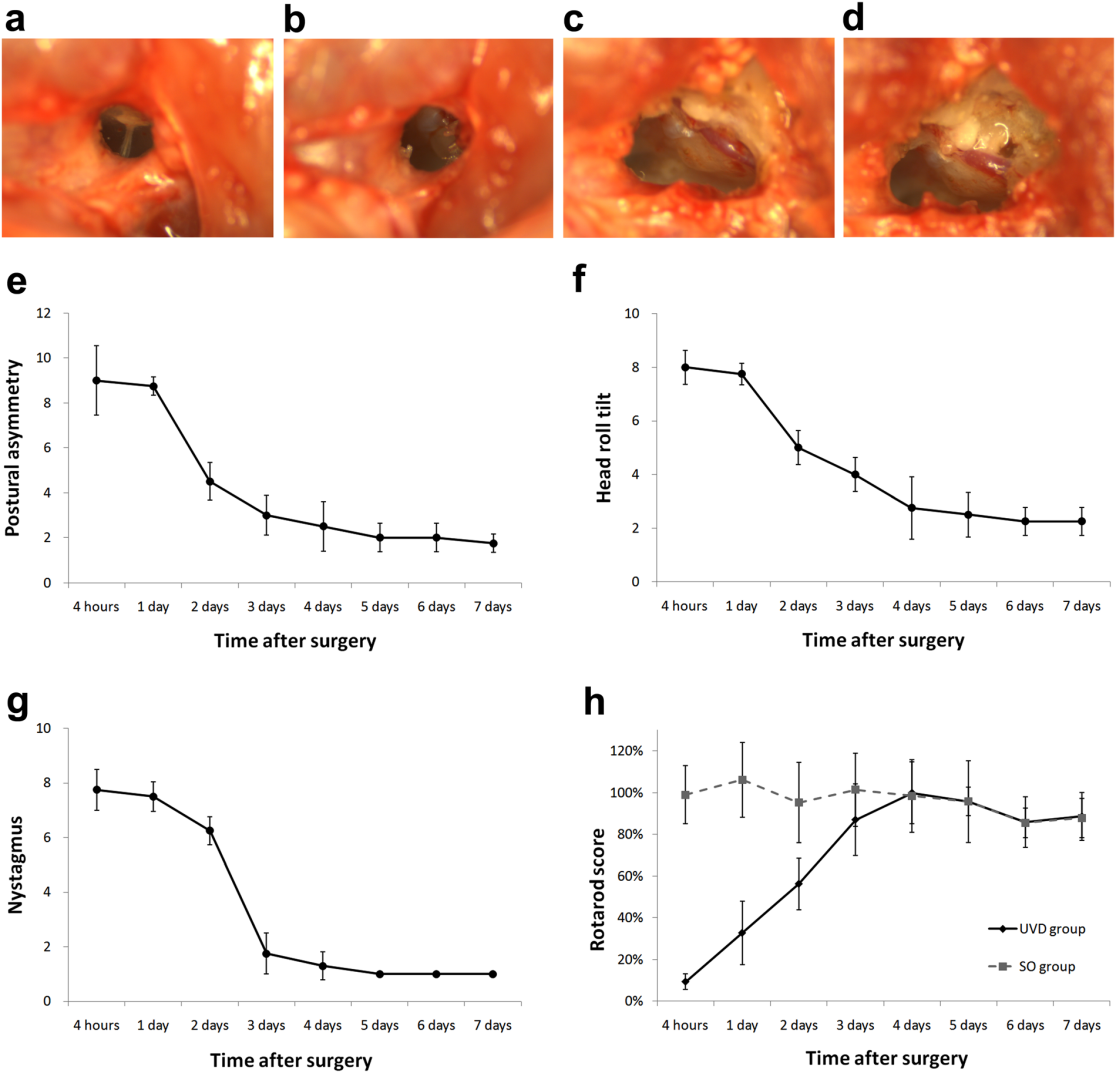
Labyrinthectomy procedure and progression of vestibular compensation after unilateral labyrinthectomy. (**a**) The tympanic membrane was exposed. (**b**) The tympanic membrane and ossicles were removed with the exception of the stapes. (**c**,**d**) The muscles on the lamboidal ridge were detached. The bone around the point of exit of the facial nerve was drilled and the bony lateral semicircular canal was exposed. Following the lateral semicircular canal anteriorly, the lateral semicircular canal was opened and the superior semicircular canal was drilled through. (**e**–**h**) Twelve SD rats were divided into two groups: the UVD group underwent unilateral labyrinthectomy (n = 6) and the SO group underwent a sham operation (n = 6). Postural asymmetry (**e**) head roll tilt (**f**) and nystagmus (**g**) were evaluated in the UVD group following surgery. Postural asymmetry and head roll tilt gradually improved over time and reached a minimum value approximately 4 d after surgery. Nystagmus significantly decreased 3 d after surgery and disappeared 4 d after surgery in almost all of the animals. (**h**) The mean rotarod scores of the UVD group increased over time following surgery and reached a maximum value 4 d after surgery. The average scores of the SO group remained steady following surgery. The error bars indicate standard error. SD, Sprague–Dawle; UVD, unilateral vestibular deafferentation; SO, sham operation.

To identify the progression of vestibular compensation following UVD, we studied two experimental groups using seven-week-old male Sprague–Dawley rats (200–250 g): the UVD group underwent unilateral labyrinthectomy (*n* = 6) (Fig. 1), and the sham operation (SO) group underwent a sham operation (*n* = 6). Both operations were performed on the left side. The behavioral scoring for vestibular deficits and the rotarod test were performed at 4 h, and 1, 2, 3, 4, 5, 6, and 7 d after surgery.

To score the vestibular deficits, postural asymmetry, head roll tilt, and nystagmus were assessed^19^. Higher scores indicate a more uncompensated state. In the UVD group, all of the animals showed severe vestibular deficits immediately following surgery. Postural asymmetry and head roll tilt gradually improved over time and reached a minimum value approximately 4 d following surgery (Fig. 1a,b). Nystagmus decreased significantly 3 d following surgery and disappeared 4 d following surgery in nearly all of the animals (Fig. 1c). The SO group showed no vestibular deficits.

Animals were trained on an automated four-lane rotarod unit (EGR FINE with 7-cm-diameter drums; Seoul, South Korea) for 10 d before surgery. The test was performed following the surgery. The latency to fall was recorded as the length of time each animal stayed on the drum. The test scores are presented as the percentage of the latency to fall during the test phase over the longest latency to fall during the training phase. The average scores of the UVD group increased over time after surgery and reached a maximum value 4 d after the surgery. The average scores of the SO group remained constant following surgery (Fig. 1d).

### Selection of candidate miRNAs using microarray analysis

Based on the results of the vestibular compensation time course, we chose two timepoints to harvest the MVNs for microarray: 4 h and 4 d after surgery. Twenty-four animals were randomly divided into one of two experimental groups: a UVD group that underwent unilateral labyrinthectomy (*n* = 12) and an SO group that underwent a sham operation (*n* = 12). Both operations were performed on the left side. Six animals from each group were anesthetized and euthanized at 4 h and 4 d following surgery. Brain tissue was harvested and the MVN on the left side was dissected. The MVNs from three animals were used for one microarray sample. Two samples were obtained for each timepoint in each group. miRNA microarrays were performed at BioCore Co., Ltd. (Seoul, South Korea) using the Agilent Rat miRNA Microarray 8 × 15 K platform.

We selected the first candidate miRNAs, which were believed to be involved in vestibular compensation, based on the results of the microarray analysis. Selection criteria were as follows. First, miRNAs in which the fold change of normalized intensity values between the two timepoints in the SO group was greater than 1.20 or less than 0.83 with *p* < 0.15 determined by Student’s *t*-test were excluded. Then, miRNAs not expressed in humans were excluded. Among the remaining miRNAs, those that satisfied one or both of the following criteria were selected: 1) fold change of the normalized intensity values between the two timepoints in the UVD group was greater than 1.30 or less than 0.77 with *p* < 0.15 determined by Student’s *t*-test, and 2) fold change of the normalized intensity values between the UVD and SO groups at the same timepoint was greater than 1.20 or less than 0.83 with *p* < 0.15 determined by Student’s *t*-test. Based on these criteria, miR-31a-5p, 133a-3p, 133b-3p, 204-5p, 206-3p, 218a-5p, 219a-5p, 221-3p, and 497-5p were selected as the first candidate miRNAs (Table 1).

**Table 1:** Candidate miRNAs selected by microarray analysis.

		Fold change	*P*-value
Fold change between 4 hours and 4 days after surgery in UL group: >1.30 or <0.77 (p < 0.15)	miR-204-5p	1.514	0.140
	miR-206-3p	1.598	0.028
	miR-218a-5p	0.708	0.064
	miR-221-3p	1.408	0.057
	miR-497-5p	1.500	0.031
Fold change between UL and SO groups 4 hours after surgery: >1.20 or <0.83 (p < 0.15)	miR-31a-5p	1.204	0.085
	miR-133a-3p	1.246	0.058
	miR-133b-3p	1.216	0.087
Fold change between UL and SO groups 4 days after surgery: >1.20 or <0.83 (p < 0.15)	miR-219a-5p	0.780	0.147

UVD, unilateral vestibular deafferentation; SO, sham operation.

### Validation of miRNA expression using qRT-PCR

**Figure 2:**
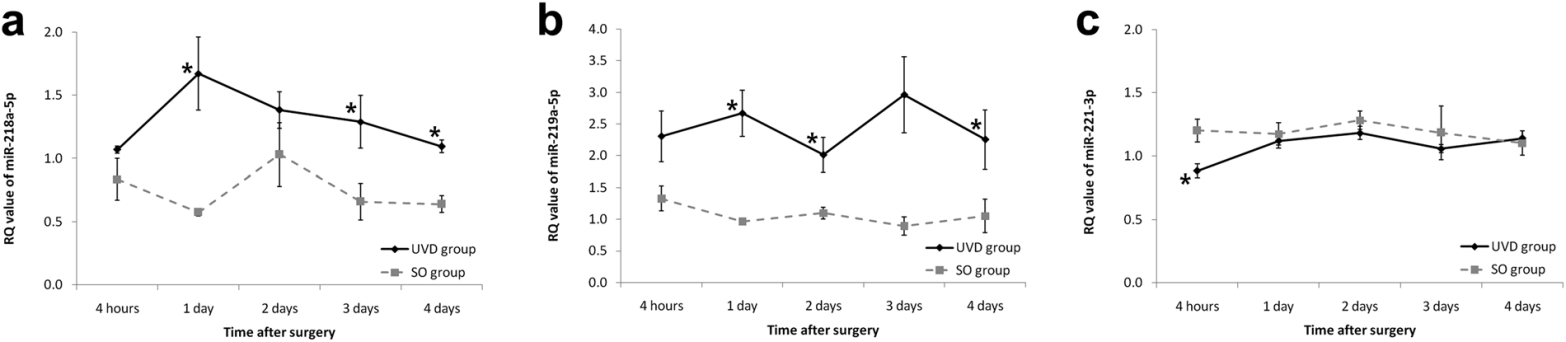
qRT-PCR of candidate miRNAs. Fifty SD rats were divided into two groups: the UVD group underwent unilateral labyrinthectomy and the SO group underwent a sham operation. MVNs were harvested at 4 h and 1, 2, 3, and 4 d following surgery. qRT-PCR was performed for miR-218a-5p (**a**) 219a-5p (**b**) and 221-3p (**c**). The differences in RQ values between the UVD and SO groups were analyzed at each timepoint. miR-218a-5p, 219a-5p, and 221-3p showed significant differences in RQ values between the UVD and SO groups at more than one timepoint. *p < 0.05 between the groups with the Mann–Whitney test. The error bars indicate standard error. qRT-PCR, quantitative reverse transcription-PCR; SD, Sprague–Dawle; UVD, unilateral vestibular deafferentation; SO, sham operation; MVN, medial vestibular nucleus; RQ, relative quantification.

To validate the first candidate miRNAs, we performed qRT-PCR. Fifty animals were randomly divided into one of two experimental groups: the UVD group underwent unilateral labyrinthectomy (*n* = 25) and the SO group underwent a sham operation (*n* = 25). Both operations were performed on the left side. Five or four animals from each group were anesthetized and euthanized at 4 h, and 1, 2, 3, and 4 d following the surgery. Five or four MVNs were obtained for each timepoint in each group and qRT-PCR was performed. The differences in relative quantification (RQ) values between the UVD and SO groups were analyzed at each timepoint using the Mann–Whitney test. miR-218a-5p, 219a-5p, and 221-3p showed significant differences in RQ values between the UVD and SO groups at more than one timepoint (Fig. 2 and Supplementary Data. 1). These miRNAs were selected as the second group of candidate miRNAs.

### Validation study using candidate miRNA oligomers

**Figure 3:**
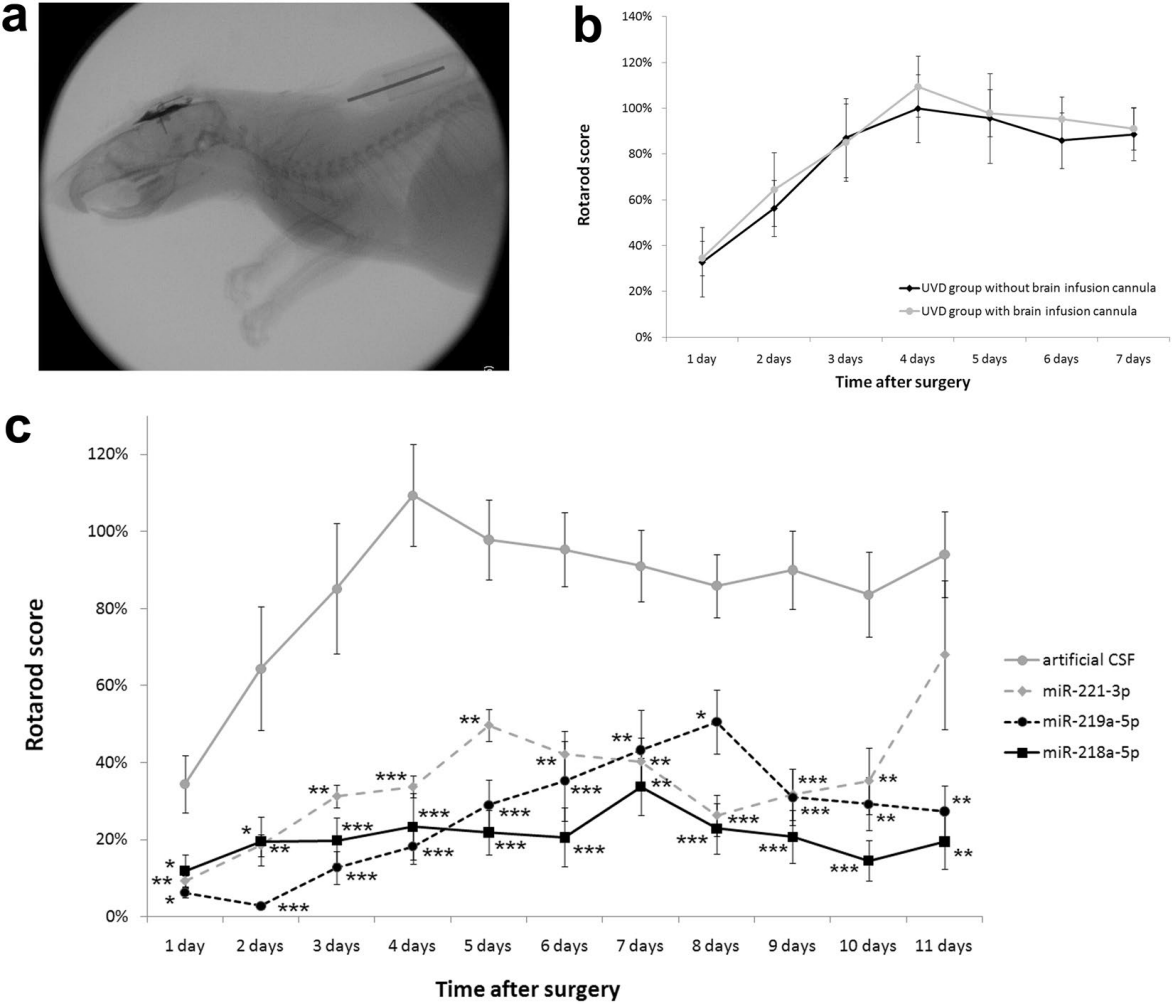
Rotarod test following administration of candidate miRNA oligomers. Twenty-four SD rats were divided into four groups. Artificial cerebrospinal fluid, miR-218a-5p antagomir, miR-219a-5p antagomir, or miR-221-3p agomir were administered into the intracerebroventricular space continuously for 1 week using the brain infusion cannula. All animals underwent unilateral labyrinthectomy 10 h following implantation of the brain infusion cannula. (**a**) X-ray image of an SD rat with implantation of the brain infusion cannula. The brain infusion cannula connected to a mini-osmotic pump was implanted into the right cerebral lateral ventricle (AP: −0.3, L: 1.2, V: 4.5) of an SD rat. (**b**) The mean rotarod scores were compared between the two groups. One group underwent unilateral labyrinthectomy without insertion of the brain infusion cannula (*n* = 6). The other group underwent unilateral labyrinthectomy with insertion of the brain infusion cannula delivering artificial cerebrospinal fluid (*n* = 6). There was no significant difference between the two groups in linear mixed models with the Bonferroni correction. (**c**) Rotarod test results following administration of candidate miRNA oligomers. The effects of the oligomers on vestibular compensation were evaluated using the rotarod test. Administration of miR-218a-5p antagomir (*n* = 6), miR-219a-5p antagomir (*n* = 6), or miR-221-3p agomir (*n* = 6) showed significant reduction in rotarod scores following surgery compared to the administration of artificial cerebrospinal fluid (*n* = 6). *p < 0.05; **p < 0.01; ***p < 0.001 (linear mixed models with the Bonferroni correction). The error bars indicate standard error. SD, Sprague–Dawle.

We performed two experiments to investigate whether administration of the second candidate miRNA oligomers (agomirs or antagomirs) affected vestibular compensation. In the case of miR-218a-5p and 219a-5p, where the candidate miRNAs were increased in the UVD group compared with the SO group, miRNA antagomirs were administered. In the case of miR-221-3p, agomirs were administered. We expected that, if the candidate miRNAs affected vestibular compensation, it would be inhibited by administration of the candidate miRNA oligomers. Thirty-six rats were randomly divided into four groups: the control group (*n* = 9), which received artificial cerebrospinal fluid (aCSF) containing (mm): 124 NaCl, 5 KCl, 1.2 KH_2_PO_4_, 1.3 MgSO_4_, 2.4 CaCl_2_, 26 NaHCO_3_ and 10 d-glucose; the 218a-5p group (*n* = 9), which received the miR-218a-5p antagomir (Qiagen, Hilden, Germany); the 219a-5p group (*n* = 9), which received the miR-219a-5p antagomir (Qiagen); and the 221-3p group (*n* = 9), which received the miR-221-3p agomir (Qiagen). aCSF and the miRNA oligomers were administered into the intracerebroventricular space continuously for one week using a brain infusion cannula (Brain Infusion Kit 2; Alzet, Cupertino, CA, USA) connected with a mini-osmotic pump (Osmotic Pump 2001; Alzet) (Fig. 3a). All animals underwent unilateral labyrinthectomy on the left side 10 h following implantation of the brain infusion cannula. Six animals in each group were used for the rotarod test and three animals were used for analysis of 5-bromo-2′-deoxyuridine (BrdU) immunohistochemistry.

The rotarod test was performed daily for 11 d following unilateral labyrinthectomy, as previously described. First, to determine whether the brain infusion cannula affected vestibular compensation, we compared the results of the rotarod test between the UVD group of a previous experiment that underwent unilateral labyrinthectomy without insertion of the brain infusion cannula and a control group of this experiment that underwent unilateral labyrinthectomy with insertion of the brain infusion cannula delivering aCSF and found there was no significant difference between the two groups (Fig. 3b). The effects of the candidate miRNA oligomers on vestibular compensation were then evaluated. The scores of the rotarod test were explored by performing linear mixed models with the treatment group and time as fixed factors, and a significant interaction between time and the treatment group (*p* < 0.001) was identified in the rotarod scores. Therefore, the primary effects for the treatment group were reported at each timepoint using linear mixed models with the Bonferroni correction. The rotarod scores of the miR-218a-5p, 219a-5p, and 221-3p groups were compared with the control group that received aCSF. The group treated with the miR-218a-5p, 219a-5p, and 221-3p oligomer showed significant reduction in rotarod scores after surgery (Fig. 3c).

**Figure 4:**
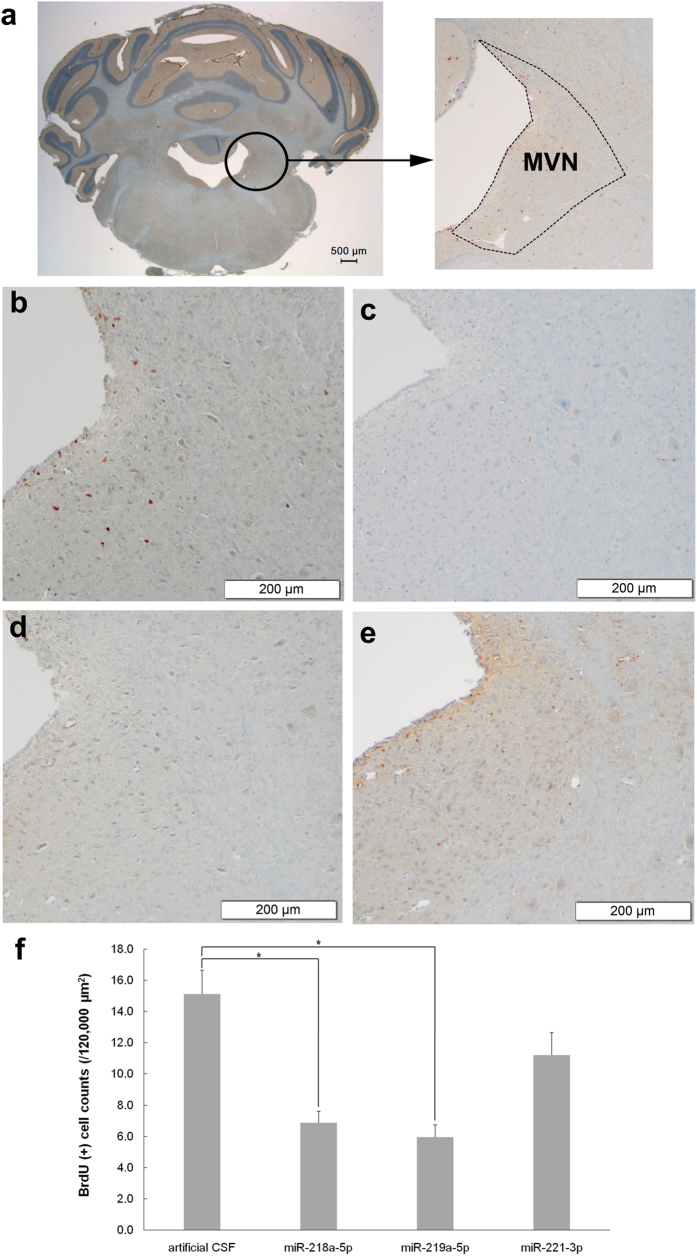
BrdU immunohistochemistry following administration of candidate miRNA oligomers. Twelve SD rats were divided into four groups. Artificial cerebrospinal fluid, miR-218a-5p antagomir, miR-219a-5p antagomir, or miR-221-3p agomir were administered into the intracerebroventricular space continuously for 1 week using the brain infusion cannula. All animals underwent unilateral labyrinthectomy 10 h following implantation of the brain infusion cannula. (**a**) MVN in the rat brain (Bregma −11.60 mm). In the animals in which artificial cerebrospinal fluid (**b**) was administered, many BrdU-positive cells were observed in the MVN. However, in the animals in which miR-218a-5p (**c**) and miR-219a-5p 5p (**d**) antagomirs were administered, very few BrdU-positive cells were observed. In the animals in which the miRNA-221-3p agomir (**e**) was administered, several BrdU-positive cells were observed in the MVN. (**f**) The mean of BrdU-positive cell counts (/120,000 μm^2^) in the MVN from each group was analyzed. BrdU-positive cell counts were significantly lower in the animals in which the miR-218a-5p (mean difference = 8.2, *p* < 0.001) and miR-219a-5p 5p (mean difference = 9.1, *p* < 0.001) antagomirs were administered compared with the control group. *p < 0.001 (one-way analysis of variance with the Bonferroni correction). The error bars indicate standard error. SD, Sprague–Dawle; MVN, medial vestibular nucleus; BrdU, 5-Bromo-2′-deoxyuridine.

In the second experiment, 300 mg/kg BrdU (Sigma, St. Louis, MO, USA) was administered by intraperitoneal injection to animals 2 d after surgery. One day later, the brains were harvested and BrdU immunohistochemistry was performed. The mean of BrdU-positive cell counts (/120,000 μm^2^) in the MVN from each group was analyzed using a one-way analysis of variance test (ANOVA) with the Bonferroni correction. The BrdU-positive cell counts were significantly lower in the 218a-5p (mean difference = 8.2, *p* < 0.001) and 219a-5p (mean difference = 9.1, *p* < 0.001) groups than in the control group. The 221-3p group had fewer BrdU-positive cells than the control group, but this difference failed to reach statistical significance (mean difference = 3.9, *p* = 0.114) (Fig. 4).

### Pathway analysis of candidate miRNA targets

**Figure 5:**
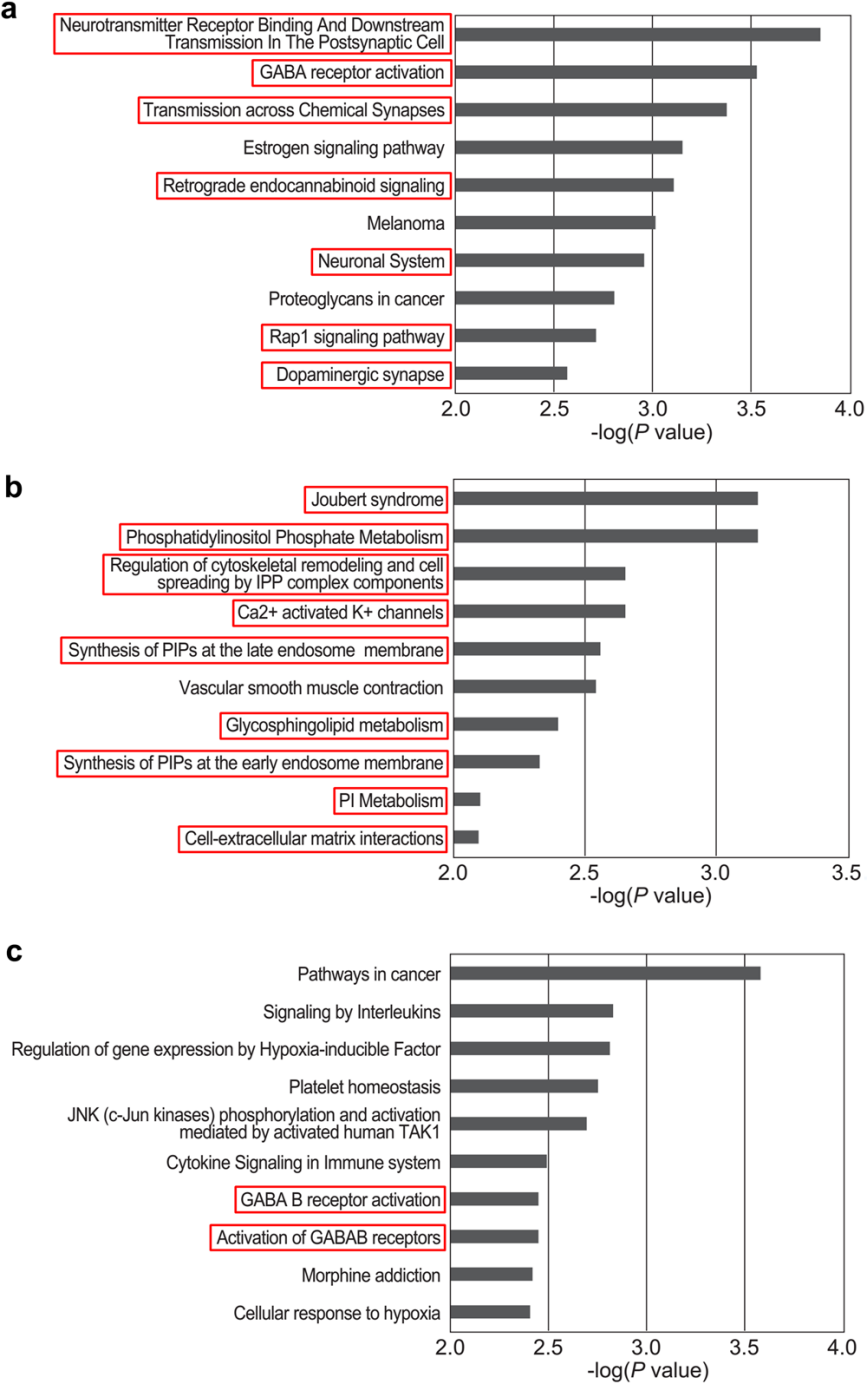
Pathway analysis of candidate miRNA targets. The ten most relevant pathways of the targets of miR-218a-5p (**a**), 219a-5p (**b**), and 221-3p (**c**) are shown. The pathways supposed to be related with vestibular compensation were highlighted with red boxes.

We also analyzed the biological pathways that may be regulated by the miRNAs identified in this study. The ten most relevant pathways of the targets of the miRNAs 218a-5p, 219a-5p, and 221-3p were determined (Fig. 5). Most of the pathways related to miR-218a-5p targets were related to signal transmission or the neuronal system. Major portions of the pathways related to miR-219-5p targets were related to intracellular signal transmission. The most relevant pathway of the targets of miR-219a-5p was related to Joubert syndrome, which exhibits a malformed brainstem and cerebellum and difficulty balancing^20^. Although the most relevant pathway of the targets of miR-221-3p was cancer, it was also related to the γ-aminobutyric acid (GABA) receptor, which is thought to be important in vestibular compensation^3^.

## Discussion and Conclusions

UVD causes disfacilitation and increased inhibition from the contralesional vestibular nuclei, leading to collapse of the resting activity of ipsilesional vestibular nuclei and an excitability imbalance between bilateral vestibular nuclei^21^. Vestibular compensation is the process through which bilateral excitability homeostasis is achieved, which overcomes this imbalance. The primary mechanism of vestibular compensation, as suggested by Precht *et al*.^1^ is a change in the reciprocal commissural inhibitory system linking the bilateral vestibular nuclei and a rebalancing of the resting activity of the vestibular nuclei. This concept has formed the basis on which studies of vestibular compensation have been conducted, and significant experimental evidence supporting this concept has accumulated^22^.

Vestibular compensation consists primarily of three neuronal mechanisms. First, the functional efficacy of neurons of the ipsilesional vestibular nuclei in response to inhibitory signals is decreased. Following UVD, GABA receptors in ipsilesional MVN neurons are rapidly downregulated^9^. Second, the intrinsic excitability of ipsilesional MVN neurons increases^7, 23^. Third, reactive neurogenesis occurs in the ipsilesional MVN. Many newly generated cells differentiate into astrocytes or GABAergic neurons, which provide commissural inhibition to contralesional vestibular nuclei neurons^24, 25^. These mechanisms reduce both initial disfacilitation and increased commissural inhibition from the contralesional vestibular nuclei neurons, which contribute to the restoration of the resting activity of the ipsilesional vestibular nuclei neurons leading to balanced activity between the two sides. However, many of the neuronal mechanisms involved in vestibular compensation have not yet been elucidated. Moreover, miRNAs related to vestibular compensation have not been reported.

miRNAs have been identified in almost all body tissues^26, 27^, and more than 1,500 miRNAs have been reported to influence the expression of 30–60% or more of all genes^27^. Furthermore, since a single miRNA can affect the expression of multiple genes, miRNAs have widespread action^12^. miRNAs have been reported to be involved in many diverse biological processes, including differentiation, apoptosis, cell fate, and tumor suppression^27, 28^. miRNAs constitute an additional layer of control within genetic networks.

Approximately 70% of all miRNAs are expressed in the nervous system^29^ and increasing evidence suggests that miRNAs are essential regulators of proper neuronal function in the mature nervous system^30–32^. Aberrant miRNA expression is associated with several neuronal diseases, such as Down’s, Rett, and fragile X syndromes, as well as autism and schizophrenia^33–35^. The role of miRNAs in the maintenance of neuronal homeostasis has also been reported. In response to the change in synaptic activity, miRNAs regulate neuronal function by controlling mRNA stability and translation^36–38^. It has been reported that miR-485 controls synapse formation and dendritic spine number in an activity-related manner to maintain homeostatic plasticity^30^. Although the mechanisms through which miRNAs regulate neuronal homeostasis are not fully understood, the morphology of dendritic spines, the expression of ion channels, and the molecular pathways downstream of receptor activation have been suggested as targets of modulation^18, 32, 39, 40^. Vestibular compensation is a phenomenon caused by an abrupt change of synaptic activity. Therefore, we hypothesized that miRNAs operate as post-transcriptional regulators of vestibular compensation and identified miRNAs that regulate vestibular compensation.

The MVN is the primary organ in which vestibular compensation occurs^3^. In the present study, we investigated the MVN to identify miRNAs involved in vestibular compensation. First, we measured vestibular compensation by scoring for vestibular deficits with the rotarod test. Vestibular compensation progressed rapidly and reached a maximum value approximately 4 d following UVD. Based on these results, microarrays were performed at two timepoints to select candidate miRNAs: when vestibular compensation had not fully progressed and when it had reached its maximal level. In addition to the comparison between the UVD and SO groups in the microarray analysis, comparison between the two timepoints in the UVD group was used for candidate selection. Criteria for the fold change and the *p*-value were as wide as 1.20 and 0.15, respectively. However, the fold change of 1.30 was used as the criterion for selecting candidate miRNAs that changed between the two timepoints in the UVD group as the fold change of 1.20 included too many candidate miRNAs. Nine miRNAs were selected as candidate miRNAs and validated by qRT-PCR. Following validation, miR-218a-5p, 219a-5p, and 221-3p were considered as the candidate miRNAs.

We then validated these miRNAs using miRNA oligomers. The agomirs or antagomirs of the candidate miRNAs were administered using a brain infusion cannula. As vestibular compensation is a rapidly progressing neural response, administration of oligomers during a short period may fail to show any effects. Therefore, we set the administration period as 7 d to identify the inhibition of vestibular compensation clearly. The concentration of oligomers was diluted to 12.5 µM according to the recommended concentration of 1–100 mM for intracerebroventricular infusion^41, 42^. The infusion rate of the mini-osmotic pump was set as 1.0 µL/h and the daily delivered dose was 24.0 µL. This was approximately 4% of 580 µL, the average cerebral spinal fluid of rats, which was set to prevent intracranial hypertension induced by intracerebroventricular infusion^43^. To evaluate the effect of each particular miRNA, the rotarod test was performed. We measured whether recovery of the rotarod scores was inhibited by administration of the candidate miRNA oligomers. In addition, neurogenesis in the MVN was evaluated. Reactive neurogenesis was reported in the ipsilesional vestibular nuclei following UVD^24, 44^. It has also been reported that behavioral recovery is inhibited when neurogenesis is prevented following UVD^24^. We measured neurogenesis with BrdU immunohistochemistry, which incorporates a thymidine analogue into newly synthesized DNA, as an indicator of vestibular compensation. Finally, the miR-218a-5p and 219a-5p groups both showed inhibited recovery of the rotarod scores and decreased neurogenesis in the MVN. In the miR-221-3p group, recovery of the rotarod scores was inhibited but neurogenesis did not decrease. Therefore, we believe that the mechanisms through which miR-218a-5p and 219a-5p affect vestibular compensation involve neurogenesis in the MVN, while miR-221-3p appears to be unrelated to neurogenesis.

Lastly, we performed pathway analysis of the targets of miR-218a-5p, 219a-5p, and 221-3p. Most of the pathways related to the targets of miR-218a-5p were involved in signal transmission or the neuronal system, suggesting that miR-218a-5p regulates vestibular compensation. Consistent with previous reports, many of the identified pathways related to the targets of miR-219a-5p were involved in intracellular signal transmission. Kocerha *et al*. (2009) reported that miR-219 was reduced in the prefrontal cortex after disruption of N-methyl-D-aspartate (NMDA)-mediated glutamate signaling, affecting the NMDA signaling cascade, which is crucial for neurotransmission and synaptic plasticity in the brain. Therefore, we expected that miR-219a-5p would alter the activity of MVN neurons. In addition, the most relevant pathway of the targets of miR-219a-5p was related to Joubert syndrome, which exhibits a malformed brainstem and cerebellum^20^. The vestibular nucleus, the primary organ where vestibular compensation takes place, is located in the brainstem. The cerebellum is necessary for increasing the excitability of MVN neurons following UVD^45, 46^. It also seems possible that miR-219a-5p regulates vestibular compensation. The most relevant pathway related to the targets of miR-221-3p was cancer-related. However, this pathway was also related to the GABA receptor, which is thought to be important in vestibular compensation^3^. Therefore, miR-221-3p may also regulate vestibular compensation. Consequently, we concluded that miR-218a-5p, 219a-5p, and 221-3p may be involved in the regulation of vestibular compensation.

## Methods

### Animals

This study and experimental protocol were approved by the Institutional Animal Care and Use Committee of Seoul National University Hospital (14-0148-C1A1), which is accredited by the Association for the Assessment and Accreditation of Laboratory Animal Care International. All experiments were performed in accordance with relevant guidelines and regulations. Each animal was acclimatized to the laboratory conditions for 1 week prior to the start of the experiment. These animals were housed in a temperature- and humidity-controlled room with a constant 12 h light: dark cycle with free access to food and water.

### Unilateral labyrinthectomy and sham operation

To generate UVD, unilateral labyrinthectomy was performed on the left side as follows (Fig. 1a–d)^47^. Animals were anesthetized by intramuscular injection of 40 mg/kg Zoletil (Zoletil 50; Virbac, Bogotá, Colombia) and by intramuscular injection of 10 mg/kg xylazine (Rompun; Bayer-Korea, Seoul, South Korea). After local anesthesia with 1% lidocaine hydrochloride, a left retroauricular incision was made to expose the external auditory canal, which was then opened to expose the tympanic membrane. The tympanic membrane and the ossicles were removed with the exception of the stapes. The muscles on the lamboidal ridge were detached. The bone around the point of exit of the facial nerve was drilled and the bony lateral semicircular canal was exposed. Following the lateral semicircular canal anteriorly, the lateral semicircular canal was opened near its ampulla. Drilling was continued in the plane of the lateral semicircular canal and the ampulla of the superior semicircular canal was drilled through. Then the contents of the vestibule were aspirated and 100% ethanol was injected and aspirated several times. In the sham operation, the same operative procedure was followed with the exception of opening the semicircular canal and injecting ethanol.

### Behavioral scoring for vestibular deficits

Behavioral scoring for vestibular deficits consisted of three components: postural asymmetry, head roll tilt, and nystagmus^19^. Each component had a maximum score of 10. The score of postural asymmetry was determined as follows: 10 points, spontaneous barrel rolling; 9 points, barrel rolling evoked by air puff or light touch; 8 points, recumbent position on the left side without leg support; 7 points, some ipsilesional leg support; 6 points, moving around on left side or using the left legs for recumbent support; 5 points, moving around with bilateral leg support; 4 points, moving around with occasional falls to the left side; 3 points, moving around leaning towards the left side; 2 points, hardly noticeable asymmetry; and 1 point, postural asymmetry only noticeable when picked up. The score of the head roll tilt was determined by measuring the angle between the jaw and the horizontal plane. A 90° angle was scored as 10 points. Cases where the animal showed barrel-rolling toward the left side or was recumbent on the left side were also scored as 10 points. The nystagmus score was determined by visual inspection. Spontaneous nystagmus was scored from 6 to 10 points, with 1 point for every 60 beats per min (bpm). When spontaneous nystagmus was absent in a resting state, the examiner gave a gentle air puff over the head of the animal. Nystagmus caused during these conditions was scored from 1 to 5 points, with 1 point for every 60 bpm.

### Rotarod test

The same protocol was applied to the training and test phase following surgery. Animals were placed on the drum that accelerated smoothly from 12 rpm with the velocity increasing by 16 rpm/min. A switch under the drum automatically measured the length of time each animal stayed on the drum. After two learning trials for habituation, three subsequent trials (T1–T3) were performed. T3 was performed 10 min after T2 for recovery. During the training period, we determined that the latency to fall reached a plateau.

### Microarray analysis

After the harvesting of brain tissue in experimental animals, the location of the MVN was identified according to the atlas of Paxinos and Watson (2006)^48^ (−10.80 mm to −12.30 mm from the bregma). The MVN on the left side was dissected, frozen in cryopreservation tubes in liquid nitrogen, and stored at −80 °C. MVNs from three animals were pooled and treated as one sample for microarray analysis. Two samples were obtained for each timepoint in one group. Microarray analysis of the miRNAs was performed at BioCore Co., Ltd. (Seoul, South Korea) using the Agilent Rat miRNA Microarray 8 × 15 K platform.

Total RNA was extracted from the sample using TRI REAGENT (MRC, Cincinnati, OH, USA), in accordance with the manufacturer’s instructions. Following homogenization, 1 mL of solution was transferred to a microfuge tube and centrifuged at 12,000 × *g* for 10 min at 4 °C to remove insoluble material. The supernatant containing RNA was collected, mixed with 0.2 mL of chloroform, and centrifuged at 12,000 × *g* for 15 min at 4 °C. After RNA in the aqueous phase had been transferred to a new tube, the RNA was precipitated by adding 0.5 mL of isopropyl alcohol and centrifuging at 12,000 × *g* for 10 min at 4 °C. The RNA pellet was washed briefly in 1 mL of 75% ethanol and centrifuged at 7,500 × *g* for 5 min at 4 °C. Finally, the RNA pellet was dissolved in nuclease-free water, and the quality and quantity of RNA were assessed with an Agilent Bioanalyzer 2100. To 500 ng of total RNA, we added calf intestinal phosphatase and T4 RNA ligase to dephosphorylate and label the 3′-end of the miRNA with Cyanine3-pCp, respectively. Labeled samples were hybridized by loading onto microarray slides using a Hybridization Chamber Kit (Agilent Technologies, Santa Clara, CA, USA) and a Hybridization Gasket Slide Kit (Agilent Technologies). Hybridization was performed over 20 h at 55 °C and the microarray slides were washed, in accordance with the manufacturer’s instructions. Processed microarray slides were scanned with an Agilent G2565CA Microarray Scanner System (Agilent Technologies). The data were processed based on the quantile normalization method using GeneSpring GX 13.1 (Agilent Technologies). This normalization method aims to make the distribution of intensities for each array in a set of arrays the same. The normalized intensity values were then analyzed and the fold changes were calculated using GeneSpring GX 13.1.

### qRT-PCR

MVNs were prepared as previously described. The MVN from one animal was placed in a tube and became one sample for qRT-PCR. Five or four samples were obtained for each timepoint in one group. The RNA was harvested using miRNeasy Mini Kit (Qiagen). cDNAs were generated using miScript II RT Kit (Qiagen). qPCR was performed in the QuantStudio 6 Flex Real-Time PCR System (Applied Biosystems, Foster City, CA, USA) using miScript SYBR Green PCR Kit (Qiagen) and miScript miRNA PCR Array (Qiagen). SNORD61 was used as an endogenous control. All PCR reactions were performed under standard PCR conditions. The cycle threshold (Ct value) was obtained. The miRNA levels of the SO group animal sacrificed 4 h postoperatively were used as controls. The data were analyzed using the 2^−ΔΔCt^ method and relative quantification values were acquired.

### Intracerebroventricular infusion of candidate miRNA oligomers

Using anesthesia conditions described previously, a brain infusion cannula connected to a mini-osmotic pump was implanted into the right cerebral lateral ventricle (AP: −0.3, L: 1.2, V: 4.5) in all the animals, according to the atlas of Paxinos and Watson (2006) (Fig. 3a). The concentration of miRNA oligomers was 12.5 µM. The infusion rate of the mini-osmotic pump was 1.0 µL/h.

### BrdU immunohistochemistry

After deep anesthesia, all animals were perfused with 0.9% saline and 4% (v/v) paraformaldehyde (PFA). The brains were removed from the skull, fixed overnight at 4 °C in PFA, and embedded in paraffin. The location of the MVN was identified according to the atlas of Paxinos and Watson (2006)^48^. Coronal sections of 10 μm were taken every 100 μm in the MVN (−11.0 mm to −12.0 mm from the bregma). Tissue sections were cut and placed on slides. Using the Discovery XT automated immunohistochemistry stainer (Ventana Medical Systems, Inc., Tucson, AZ, USA), slides were stained as follows. Detection was performed using the Ventana Chromo Map Kit (Ventana Medical Systems, Inc.). Sections were deparaffinized using EZ Prep solution. CC1 standard (Tris/Borate/EDTA buffer pH 8.4) was used for antigen retrieval. Inhibitor D (3% H_2_O_2_, endogenous peroxidase) was blocked for 4 min at 37 °C. Slides were incubated with the primary monoclonal mouse anti-BrdU antibody (1:100; Dako, Santa Clara, CA, USA) for 32 min at 37 °C, and the secondary Discovery UltraMap anti-mouse horseradish peroxidase antibody (Ventana Medical Systems, Inc.) for 20 min at 37 °C. Slides were incubated in DAB + H_2_O_2_ substrate for 8 min at 37 °C followed by hematoxylin and bluing reagent counterstain at 37 °C. Reaction buffer (Tris buffer, pH 7.6) was used as the washing solution.

The number of BrdU-positive cells was counted under a light microscope (CX31; Olympus, Tokyo, Japan). Ten sequential sections of the MVN on the left side in 100-μm steps were evaluated. BrdU-positive cells were counted using an integrated microscopic counting chamber that delineated the region of interest by a square of 40,000 μm^2^. The sum of BrdU-positive cell counts from the three regions of interest (/120,000 μm^2^) was obtained for each section. The average cell counts of BrdU-positive cells from ten sections were used for statistical analysis.

### Target pathway analysis of candidate miRNAs

The list of predicted targets was downloaded from the TargetScan (v7.1) web-server (http://www.targetscan.org/). We selected all predicted targets irrespective of site conservation. Using the mRNA expression profiles for the vestibular nucleus samples with similar timepoints that we obtained previously^49^, we only selected mRNAs that showed an inverse correlation with the expression profile of each miRNA. For the filtered mRNAs, we performed overrepresentation analysis with ConsensusPathDB (http://cpdb.molgen.mpg.de/). Among the pathways identified, we selected only those from Reactome, the Kyoto Encyclopedia of Genes and Genomes, and the Small Molecule Pathway Database. Based on the *p* value, we selected the top 10 pathways.

### Statistical analyses

IBM SPSS software version 21.0 (IBM, NY, USA) was used for statistical analyses.

## Electronic supplementary material

Quantitative reverse transcription-PCR (qRT-PCR) of candidate miRNAs
