# Supplementary material for: MicroRNAs 218a-5p, 219a-5p, and 221-3p regulate vestibular compensation
Source: Sci Rep. 2017 Aug 18;7:8701. doi: 10.1038/s41598-017-09422-8 (PMC5562769; doi:10.1038/s41598-017-09422-8)

[Supplementary Information]

MicroRNAs 218a-5p, 219a-5p, and 221-3p regulate vestibular compensation

Mun Young Chang[1,2], Sohyeon Park[2], Jun Jae Choi[2], Young-Kook Kim[3], Myung-Whan Suh[2], Jun Ho Lee[2], Seung Ha Oh[2] & Moo Kyun Park[2,*]

[1]Department of Otorhinolaryngology-Head and Neck Surgery, Chung-Ang University College of Medicine, Seoul, 06973, Republic of Korea. [2]Department of Otolaryngology-Head and Neck Surgery, Seoul National University Hospital, Seoul National University College of Medicine, Seoul, 03080, Republic of Korea. [3]Department of Biochemistry, Chonnam National University Medical School, Gwangju, 61186, Republic of Korea.

***Correspondence and requests for materials should be addressed to**: Moo Kyun Park, MD, PhD. Department of Otorhinolaryngology-Head and Neck Surgery, Seoul National University College of Medicine, 101 Daehak-Ro Jongno-Gu, Seoul 110-744, Republic of Korea

Tel; 82-2-2072-2446, Fax:82-2-745-2387

E-mail: aseptic@snu.ac.kr (M.K.P.)

Supplementary Data 1. Quantitative reverse transcription-PCR (qRT-PCR) of candidate miRNAs. Fifty SD rats were divided into two groups: the UVD group underwent unilateral labyrinthectomy and the SO group underwent a sham operation. MVNs were harvested at 4 h, and 1, 2, 3, and 4 d after surgery. qRT-PCR was performed for miR-31a-5p (a), 133a-3p (b), 133b-3p (c), 204-5p (d), 206-3p (e), and 497-5p (f). The differences in the RQ values between the UVD and SO groups were analyzed at each timepoint. These miRNAs showed no significant differences in RQ values between the UVD and SO groups by the Mann–Whitney test. The error bars indicate standard error.

UVD, unilateral vestibular deafferentation.

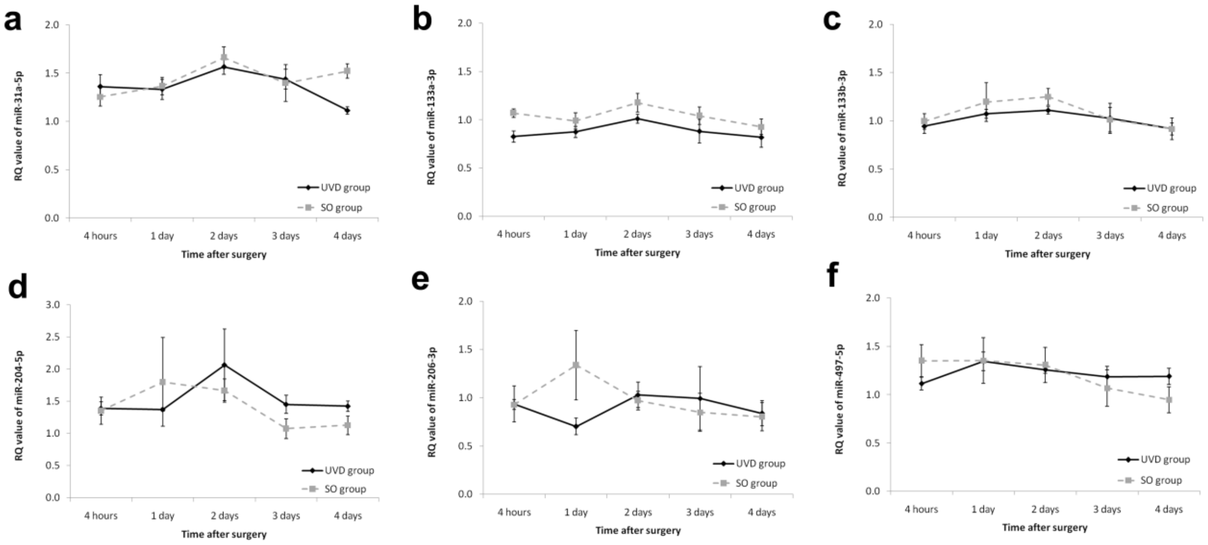

Supplement: Supplementary file 1 — Quantitative reverse transcription-PCR (qRT-PCR) of candidate miRNAs [file 41598_2017_9422_MOESM1_ESM.pdf]
